# Graphene Oxide Nanoparticles Modified Paper Electrode as a Biosensing Platform for Detection of the *htrA* Gene of *O. tsutsugamushi*

**DOI:** 10.3390/s21134366

**Published:** 2021-06-25

**Authors:** Deepak Kala, Tarun Kumar Sharma, Shagun Gupta, Vivek Verma, Atul Thakur, Ankur Kaushal, Alex V. Trukhanov, Sergei V. Trukhanov

**Affiliations:** 1 Amity Center of Nanotechnology, Amity University, Gurugram 122413, India; deepak.kala@student.amity.edu (D.K.); athakur1@ggn.amity.edu (A.T.); 2 Translational Health Science and Technology Institute, Faridabad 121001, India; tarun@thsti.res.in; 3 School of Bioengineering and Food Technology, Shoolini University, Solan 173229, India; shagungupta@shooliniuniversity.com (S.G.); vivekverma@isrsindia.co.in (V.V.); 4 Department of Technology of Electronics Materials, National University of Science and Technology “MISiS”, Leninsky Av., 4, 119049 Moscow, Russia; truhanov86@mail.ru; 5 Laboratory of Magnetic Films Physics, SSPA “Scientific and Practical Materials Research Centre of NAS of Belarus”, 19 P. Brovki str., 220072 Minsk, Belarus; 6 Laboratory of Single Crystal Growth, South Ural State University, 76, Lenin Av., 454080 Chelyabinsk, Russia

**Keywords:** scrub typhus, screen printed paper electrode, DNA sensor, electrochemical, *htrA* gene

## Abstract

The unique structural and electrochemical properties of graphene oxide (GO) make it an ideal material for the fabrication of biosensing devices. Therefore, in the present study, graphene oxide nanoparticles modified paper electrodes were used as a low-cost matrix for the development of an amperometric DNA sensor. The graphene oxide was synthesized using the modified hummers method and drop cast on a screen-printed paper electrode (SPPE) to enhance its electrochemical properties. Further, the GO/SPPE electrode was modified with a 5′NH2 labeled ssDNA probe specific to the *htrA* gene of *Orientia tsutsugamushi* using carbodiimide cross-linking chemistry. The synthesized GO was characterized using UV-Vis, FTIR, and XRD. The layer-by-layer modification of the paper electrode was monitored via FE-SEM, cyclic voltammetry, and electrochemical impedance spectroscopy (EIS). The sensor response after hybridization with single-stranded genomic DNA (ssGDNA) of *O. tsutsugamushi* was recorded using differential pulse voltammetry (DPV). Methylene blue (1 mM in PBS buffer, pH 7.2) was used as a hybridization indicator and [Fe(CN)_6_]^−3/−4^ (2.5 mM in PBS buffer, pH 7.2) as a redox probe during electrochemical measurements. The developed DNA sensor shows excellent sensitivity (1228.4 µA/cm^2^/ng) and LOD (20 pg/µL) for detection of *O. tsutsugamushi* GDNA using differential pulse voltammetry (DPV).

## 1. Introduction

Scrub typhus, one of the most neglected and re-emerging infectious diseases of the tsutsugamushi triangle, is caused by a Gram-negative bacterium known as *Orientia tsutsugamushi* [1,2]. The disease is highly prevalent in rural areas, where proper diagnosis facilities are not available to detect the infection at early stages resulting in higher mortalities. The clinical symptoms of the disease are very common and similar to the other infections that make it difficult to diagnose [1,3]. The bacteria spread through the infected larval mite that produces eschar at the biting site. The diversified distribution of eschar among the scrub typhus patients makes it an unsuitable marker for initial screening [1,4]. Continuously used expand methods for diagnosing scrub typhus include the immunochromatographic test (ICT), enzyme-linked immunosorbent assay (ELISA), and immunofluorescence assay (IFA). These methods are sensitive and specific for the detection of antibody titer but only up to a certain detectable level. However, these are not able to detect the infection at early stages [1,3]. The molecular assay, such as RT-PCR, is sensitive and specific but shows false-negative results owing to the diversified genetic makeup of *O. tsutsugamushi* and lower recovery of its genomic DNA (GDNA) from the patient’s blood samples [3]. The drawback of existing diagnosis methods (serological and molecular) triggers the need for an ideal sensing platform for the detection of *O. tsutsugamushi* with higher sensitivity, specificity, and at early stages of disease development.

Low-cost disposable materials, such as paper-based electrodes, have gained considerable attention for the construction of electrochemical sensors due to their ability to work with low sample volume, low cost, and higher flexibility [5,6,7,8,9,10,11]. These electrodes show excellent sensitivity, LOD, and stability in the detection of the targeted analyte using different samples [12,13,14,15,16]. The performance of such electrodes in terms of sensitivity and stability can be polished using different nanomaterial and their composites. The paper electrodes modified using carbon-based nanomaterials have gained substantial attention due to the ease of coating and printing to improve the performance of the biosensor [11]. The use of a graphene oxide nanomaterial for electrochemical biosensing has gained provenance of excellent performance in electrocatalysis and lower resistance to charge transfer ratio [17]. The GO contains oxygen functional groups, such as hydroxyl, carboxylic acids, and epoxy, that show good catalytic activity. They aid in facile surface modification with the desired biomolecule (bioreceptors) using different cross-linking chemistry [18]. Graphene oxide has been used in the development of various electrochemical biosensors and proved to be an excellent and economical nanomaterial for biosensing applications [19,20].

There are several applications that utilize GO nanomaterials and their nanocomposites for the development of DNA sensors using different matrices, such as pencil graphite electrodes and glassy carbon electrodes [21,22,23,24]. Keeping in view the advantages of GO in biosensing applications, a simple approach was used to develop a low-cost point-of-care system using screen-printed paper electrodes. No DNA biosensor has been developed so far using such an approach with high sensitivity and a lower limit of detection. The method was based on electrochemical detection using a screen-printed paper electrode modified with graphene oxide and ssDNA probe specific to the *htrA* gene of *O. tsutsugamushi.* The *htrA* gene sequence was retrieved from NCBI, and its specificity to the targeted organism was evaluated using alignment tools (BLAST, CLUSTAL W).

**Scheme 1:**
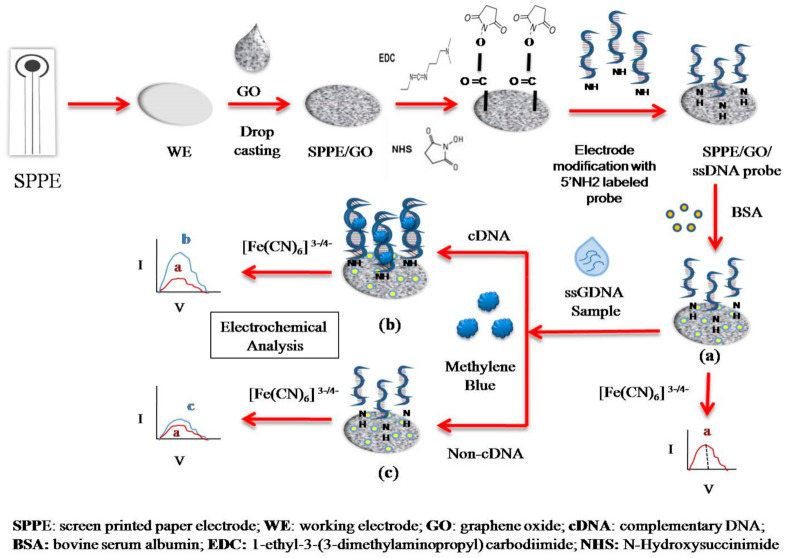
Schematic representation of SPPE/GO/ssDNA_probe_/BSA fabrication process with assay protocol for detection of *O. tsutsugamushi.*

The *htrA* gene shows homology only with the targeted organism that ensured its specificity. The method was based on the principle of nucleic acid hybridization, and methylene blue (MB) dye was used as a hybridization indicator. The readings were taken using potassium ferricyanide as a redox probe. The MB intercalates with the DNA bases in oxidized form. Upon applying potential on the electrode surface, MB was reduced to LB (leucomethylene blue) via DNA-mediated charge transfer. LB has less efficiency for DNA and dissociates and reoxidized to MB by reducing the ferricyanide to ferrocyanide freely diffusing in the solution. The regenerated MB intercalated with the DNA bases and completed the catalytic cycle. The potassium ferricyanide acted as an electron sink and the ferrocyanide in the solution reoxidized to the MB on the electrode surface. This electrocatalysis results in increased current magnitude in comparison to those produced by direct electrochemical reduction of MB [25]. The DNA sensor fabrication process and the assay protocol for the detection of *O. tsutsugamushi* are illustrated in Scheme 1.

## 2. Experimental

### 2.1. Materials

Sodium nitrate (NaNO_3_), sulfuric acid (H_2_SO_4_)_,_ potassium permanganate (KMnO_4_) were purchased from Alkem Laboratories, Lower Parel, India. The methylene blue, potassium ferricyanide/ferrocyanide, and graphite powder were purchased from Loba Chemie, Mumbai, India. EDC and NHS were purchased from Sigma Aldrich, St. Louis, USA. The chemicals for preparation of PBS buffer, pH 7.2 (0.137 M NaCl, 0.0027 M KCl, 0.01 M Na_2_HPO_4_, and 0.0018 M NaH_2_PO_4_) and TE buffer, pH 8.0 (10 mM Tris and 1 mM EDTA) were procured from Qualigens, India. The single-stranded DNA probe (ssDNA_probe_) 21 mer (5′NH_2_ TGGGGTCTTGGATTCTTGTGA 3′) was synthesized from BioServe Biotechnologies (India) Pvt. Ltd. All reagents and buffer solutions were prepared using milliQ water. The GDNA from the patient’s blood samples and other bacterial cultures (*K. pneumoniae*, *L. interrogans*) were isolated using QIA amp DNA mini kit. The screen-printed paper electrodes were procured from Class One systems Pvt. Ltd., New Delhi, India and modified at the biosensor technology lab, Amity University, Gurugram, Haryana, India.

### 2.2. Apparatus

Electrochemical measurements in terms of differential pulse voltammetry (DPV), cyclic voltammetry (CV), and electrochemical impedance spectroscopy (EIS) were performed using Palmsense4 potentiostat/Galvanostat. The screen-printed paper electrodes (class one systems, New Delhi, India) were used as a sensing platform and modified at the biosensor technology laboratory, Amity University, Haryana, India. The GO were characterized using UV-Vis spectra (Agilent, Santa Clara, CA, USA), FTIR (Perkin Elmer, Frontier, New Delhi, India), XRD (Rigaku, Wilmington, NC, USA), and FE-SEM (Carl Zeiss Ultra Plus, Jena, Germany).

### 2.3. Synthesis of Graphene Oxide

The graphene oxide nanoparticles were synthesized using the hummers method [26]. The graphite powder (0.625 g) was mixed with sodium nitrate (0.47 g), and 46.9 mL concentrated sulfuric acid was added slowly to the flask. The solution was mixed properly by stirring at lower temperature (room temperature), and potassium permanganate (1.88 g) was added in portions to the solution carefully. The solution was continuously stirred at room temperature (RT) for the next 5 days and then treated with 125 mL of an aqueous solution of H_2_SO_4_ (5%). The solution was heated at 98 °C for another 2 h, followed by cooling to 60 °C thereafter 3.75 mL 30% H_2_O_2_ was added to it, and it was again heated at 98 °C for 2 h. The resulting solution was then centrifuged at 10,000 rpm for 30 min to separate out the pellet. The pellet was washed with 5% H_2_SO_4_ (10 times), 5% HCl solution (5 times), followed by multiple washing steps using distilled water until the pH of the supernatant became neutral. The obtained pellets were dried overnight in a vacuum oven to obtain graphene oxide nanoparticles.

### 2.4. Modification of the Screen-Printed Paper Electrode (SPPE) Using GO (SPPE/GO)

The screen-printed paper electrode (SPPE) consisting of a working electrode (carbon), counter electrode (carbon), and reference electrode (Ag/AgCl) was used as a matrix for the development of the electrochemical DNA sensor. The WE surface was modified with GO nanoparticles using the drop casting method. The working electrode (WE) surface was washed with ethanol, followed by distilled water to remove the adsorbed impurities from the surface. Then GO (1 mg) was dissolved in DMSO solution (1 mL) and sonicated at 40 °C for 20 min. This suspension (3 µL) was added onto the WE and dried at 40 °C. The electrode was then washed with distilled water to remove the unbounded material from the surface.

### 2.5. Fabrication of the DNA Sensor (SPPE/GO/ssDNA_probe_/BSA)

The GO modified screen-printed paper electrode (SPPE/GO) was further modified with a 5′NH_2_ linked ssDNA probe specific to the *htrA* gene of *O. tsutsugamushi*. The ssDNA probe (20 pmol in TE buffer, pH 8.0) was covalently attached to the modified electrode surface by coupling carboxyl groups of GO with amine groups of the ssDNA probe using EDC/NHS (1 mM each). The SPPE/GO working electrode was treated with 3 μL EDC/NHS (1:1) solution prepared in PBS buffer, pH 7.2, and incubated for 90 min at RT. The electrode was then washed with PBS buffer and dried at RT followed by the addition of the ssDNA probe (3 μL). This electrode was then incubated overnight at 7–8 °C in a humid chamber, followed by multiples washing using TE buffer (pH 8). The exposed area of the working electrode was covered by treating the WE electrode with 0.5% BSA solution (3 μL) for 60 min. Then the electrode was washed using TE buffer and stored at 4 °C in a humid chamber until further use.

### 2.6. Characterization

The GO were characterized using UV-Vis, FTIR, and XRD in order to study the morphology and functional groups of nanoparticles. Further, the topographies of bare SPPE, SPPE/GO, and SPPE/GO/ssDNA_probe_ were delineated using FE-SEM. The electrode at each step of fabrication was monitored using cyclic voltammetry (CV) and electrochemical impedance spectroscopy (EIS) using [Fe(CN)_6_]^−3/−4^ (2.5 mM, PBS buffer pH 7.2) as a redox probe. The CV was recorded in a potential range of −0.5 V to 0.7 V with a scan rate of 30 mV/s. The EIS spectra were recorded in a frequency range of 0.1 Hz to 10^5^ Hz with the Edc = 0.15 V and Eac = 0.006 V. The electrochemical studies were performed in order to delineate the fabrication steps as well as the hybridization event on the electrode surface.

### 2.7. Measurement of DNA Sensor Response

The SPPE/GO/ssDNA_Probe_/BSA was hybridized with different dilutions of *O. tsutsugamushi* ssGDNA. The ssGDNA was mixed with MB dye (1 mM) in 1:1 and added to the WE surface for 10 min at RT in a humid chamber. The electrode was washed multiple times with PBS buffer, followed by TE buffer to remove unhybridized ssGDNA from the electrode surface. After hybridization, the electrochemical measurements using differential pulse voltammetry (DPV) were performed in the presence of [Fe(CN)_6_]^−3/−4^ as a redox probe. The sensitivity (S) was calculated using the formula S = m/A, where “m” is the slope of the linear equation and “A” is the area of the working electrode. The limit of detection (LOD) was calculated by dividing the standard deviation of the average current values by the sensitivity, LOD = σ/S (σ = standard deviation, S = sensitivity).

### 2.8. Selectivity, Specificity and Stability

The selectivity of the DNA sensor was evaluated with the ssGDNA of *O. tsutsugamushi* and other bacterial species, such as *K. pneumoniae*, *L. interrogans*, TE buffer without GDNA, and human GDNA. Similar concentrations (20 ng/μL) of all ssGDNA samples were hybridized onto the DNA sensor, and the response was measured using DPV in order to evaluate the selectivity towards the targeted organism. The DNA sensor selectivity and specificity were also evaluated using cDNA sequences with different numbers of mismatched bases. The stability of SPPE/GO/ssDNA_Probe_/BSA was evaluated using CV in the presence of ssGDNA of *O. tsutsugamushi.*

## 3. Results and Discussion

### 3.1. Characterization of GONPs

**Figure 1:**
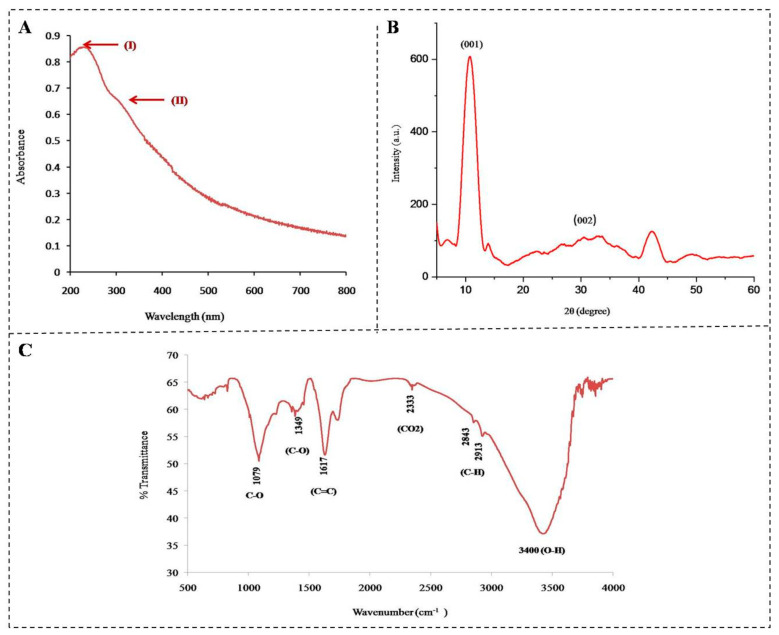
(**A**–**C**) Characterization of graphene oxide using UV-Vis (**A**), FTIR (**B**), and XRD (**C**). The UV-Vis spectra were obtained in wavelengths of 200 to 800 nm, and the FTIR spectra in a frequency range of 500 to 4000 cm^−1^.

Figure 1A shows the UV-Vis spectrum of graphene oxide. The spectrum exhibited a sharp absorption peak at 228 nm (I) due to π-π* transition of aromatic C-C bonds, and an additional shallow peak at 270–334 nm (II) corresponded to n-π* transition of C=O [27,28,29]. The abundance of aromatic C-C bonds is due to the oxidation process of graphite that results in the formation of GO. The additional peak manifests the abundance of n-π* transition of the carbonyl groups.

Figure 1B shows the FTIR spectrum of graphene oxide prepared by the Hummers method. The FTIR spectrum was in good agreement with previous work [28,30,31,32,33]. The spectrum exhibited peaks corresponding to different functional groups containing an oxygen configuration in their structures, such as stretching vibrations of the OH group (3400 cm^−1^), which may be from intercalated water molecules or phenol or of carboxyl groups [28,30]. The peaks at 2843 and 2913 cm^−1^ are of the OH stretching of alcohols and H bonded OH groups of dimeric COOH groups. The peaks at 1715 cm^−1^, 1079 cm^−1^, 1617 cm^−1^ were of C=O (carboxylic groups), C-O (alkoxy) stretching vibration, and sharp C=C band, respectively [31,32].

XRD analysis was also performed to resolve the average crystallite structural properties of graphene oxide. The XRD pattern was in agreement with the previous studies [34,35]. The XRD pattern of GO in Figure 1C shows a sharp peak at an angle (2θ) = 10.74° assign to (001) graphene oxide. The shallow peak at an angle (2θ) = 29.4° (002) is attributed to graphite which is shifted to 10.74° after oxidization of graphite to GO [35]. A broad peak at a range of 11° to 28° might be the sign of incomplete oxidation of graphite.

The crystalline grain size (D) of GO was calculated using the Debye Scherrer equation [36]

$$D=\frac{0.9\lambda }{\beta cos\theta \;}$$

where *λ* = 0.154 nm, *β* = FWHM in radians and, *θ* = location of peak in radians.

The crystalline grain size of GO using the obtained XRD pattern was calculated as 4.29 nm. The XRD results ensured the successful synthesis of GO.

### 3.2. Surface Characterization

**Figure 2:**
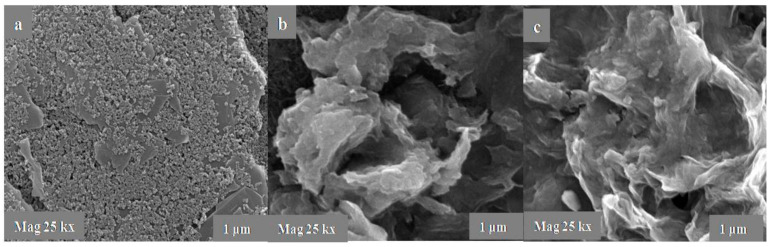
FE-SEM images of (**a**) bare screen-printed paper electrode (SPPE), (**b**) SPPE modified with graphene oxide (SPPE/GO), (**c**) 5′NH_2_ ssDNA_probe_ modified SPPE/GO (SPPE/GO/ssDNA_probe_).

FE-SEM was used to study the morphological changes of the modified SPPE at each step of fabrication (Figure 2). Figure 2a illustrates the image of bare SPPE that contained a uniform layer of carbon-based material. After the drop casting of GO onto the SPPE surface the resulting FE-SEM image (Figure 2b) showed changes in surface morphology due to the covering of electrode surface by graphene oxide. The 5′NH_2_ ssDNA probe was immobilized onto the SPPE/GO using carbodiimide cross-linking chemistry, and the modified electrode (SPPE/GO/ssDNA_probe_) was imaged. The FE-SEM images obtained after modification of SPPE/GO with ssDNA_probe_ indicate clearly that the immobilization process made electrode surface (SPPE/GO/ssDNA_probe_) cloudy and denser than the previous step. Further formation of the biomolecule layer (ssDNA_probe_) onto the modified electrode surface (SPPE/GO) is attributed to the changes in surface morphology, as shown in Figure 2c.

### 3.3. Electrochemical Characterizations

**Figure 3:**
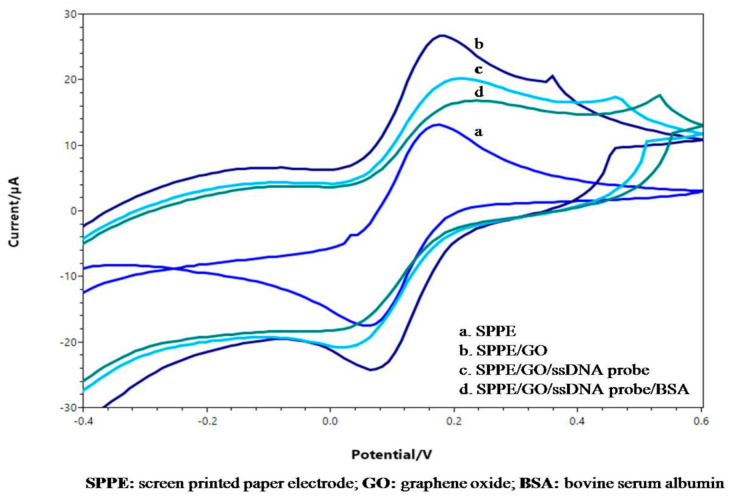
CV of (**a**) SPPE, (**b**) SPPE/GO, (**c**) SPPE/GO/ssDNA_probe_, (**d**) SPPE/GO/ssDNA_probe_/BSA. The readings were recorded using 2.5 mM [Fe(CN)_6_]^3−/4−^ prepared in PBS buffer, pH 7.2; scan rate 30 mV/s.

The step-by-step changes in SPPE surface properties were evaluated by CV and EIS, using potassium ferricyanide (2.5 mM) prepared in PBS buffer, pH 7.2. Figure 3 shows a cyclic voltammogram of the SPPE electrode at different steps of modifications. Figure 3 curve a shows a well-defined redox peak of bare SPPE using [Fe(CN)_6_]^3−/4−^. The modification of SPPE with GO (SPPE/GO) enhanced the surface area, and the conductivity of the electrode surface resulted in elevated current value (curve b). Immobilization of ssDNA_probe_ onto the SPPE/GO surface caused a reduction in the current of the [Fe(CN)_6_]^3−/4−^ (curve c). The reduction in the current value ensured the attachment of ssDNA_probe_ onto the electrode surface, and the decrement in current was attributed to the negatively charged phosphate group of DNA that repelled the [Fe(CN)_6_]^3−/4−^. The blockage of the exposed electrode surface (SPPE/GO/ ssDNA_probe)_ using BSA caused the reduction in the current value due to the insulating layer of biomolecule formed on the electrode surface (curve d).

**Figure 4:**
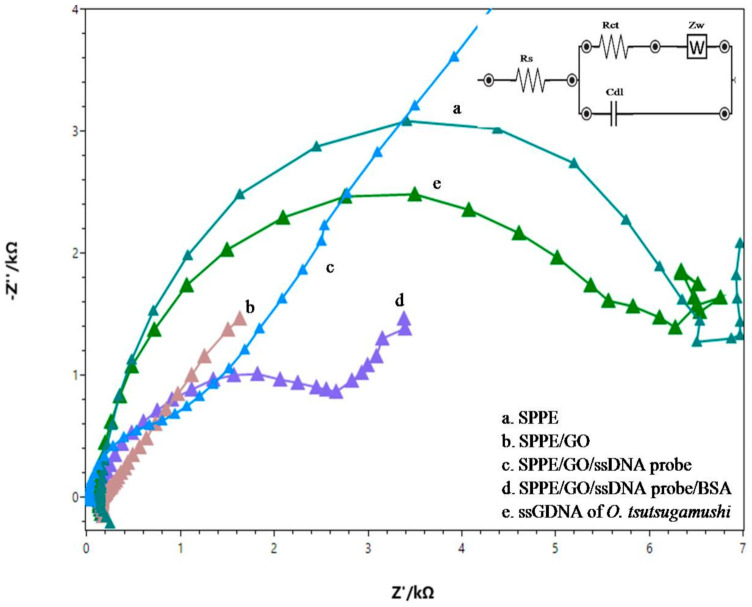
EIS spectra of (**a**) SPPE, (**b**) SPPE/GO, (**c**) SPPE/GO/ssDNA_probe_, (**d**) SPPE/GO/ssDNA_probe_/BSA, (e) after hybridization with ssGDNA of *O. tsutsugamushi*. The readings were recorded using 2.5 mM [Fe(CN)_6_]^3−/4−^ prepared in PBS buffer, pH 7.2; frequency range of 0.1 Hz to 10^5^ Hz with the Edc = 0.15 V and Eac = 0.006 V.

An EIS was also performed to confirm the SPPE/GO/ssDNA_probe_ fabrication. Figure 4 shows EIS spectra of (a) bare SPPE, (b) SPPE/GO, (c) SPPE/GO/ssDNA_probe_ and, (d) hybridization with ssGDNA of *O. tsutsugamushi*.

The Randles equivalent circuit (Figure 4 inset) was used for curve fitting and to evaluate the electron transfer resistance (Rct) values of experimental data.

As shown in Figure 4 curve b, the Rct value of SPPE/GO (Rct = 0.019 kΩ) was found to be lower than the bare SPPE (Figure 4, (curve a) Rct = 6.12 kΩ). The decrease in electron transfer resistance was attributed to the modification of SPPE by GO that enlarged the surface area and enhanced the electron transfer on electrode surface. In the next step, the attachment of ssDNA_probe_ onto the SPPE/GO further increased the Rct = 0.91 kΩ value (Figure 4 curve c), indicating the hindrance in electron transfer on the electrode surface. The increase in Rct value resulted from the negatively charged phosphate group of DNA that caused repulsion to the redox probe, [Fe(CN)_6_]^3−/4−^. Further covering of the exposed electrode surface by BSA caused the blockage of the electrode surface and, consequently, Rct = 2.12 kΩ increased (Figure 4 curve d).

Incubation of ssGDNA of *O.tsutsugamushi* with the modified electrode surface (SPPE/GO/BSA/ssDNA_probe)_ further resulted in an increase in Rct value, i.e., 5.56 kΩ (Figure 4 curve e), indicating the successful hybridization of targeted DNA with the probe on the electrode surface. The EIS responses at different stages showed that the SPPE/GO/BSA/ssDNA_probe_ was fabricated successfully.

### 3.4. Performance of Electrochemical DNA Sensor

**Figure 5:**
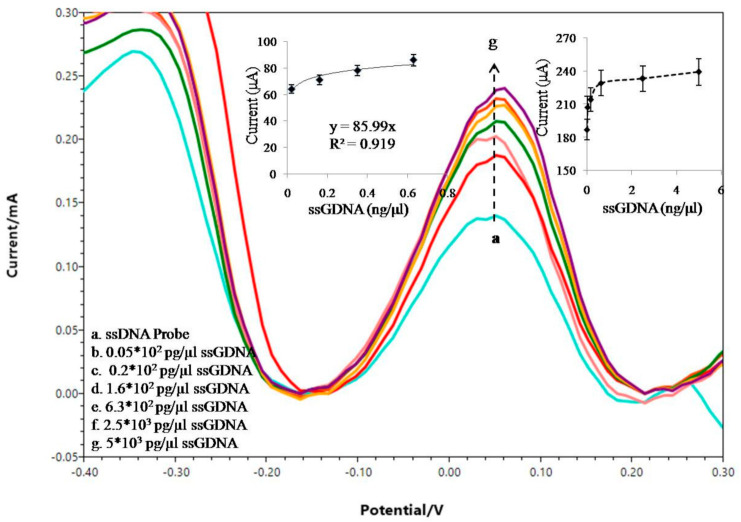
DPVs obtained after hybridization with different concentrations of *O. tsutsugamushi* GDNA ranging from 0.05 × 10^2^ pg/µL to 5 × 10^3^ pg/µL using 1 mM methylene blue (prepared in PBS buffer, pH 7.2) as a hybridization indicator and 2.5 mM [Fe(CN)_6_]^3−/4−^ as a redox probe; scan rate 30 mV/s. The inset shows the standard calibration curve obtained by plotting Ip vs. concentrations of DNA and used for calculation of sensitivity and LOD.

The SPPE/GO/BSA/ssDNA_probe_ was incubated for 10 min with different concentrations (0.05 × 10^2^ to 2.5 × 10^3^ Pg/µL) of heat-denatured (95 °C for 5 min.) GDNA of *O. tsutsugamushi* to evaluate the performance of the developed sensor (Figure 5). The electrochemical response was recorded via DPV.

An increase in the peak current was recorded with an increasing concentration of *O. tsutsugamushi* GDNA on the electrode surface. This was due to the attractive forces between positively charged MB molecules, which intercalated to the DNA bases and the redox probe [Fe(CN)_6_]^3−/4−^. The ssGDNA of *O. tsutsugamushi* was hybridized to the sensor surface along with the methylene blue that has binding efficiency with the DNA bases [37] therefore speeding up the hybridization process. The MB bonded to the DNA bases enhanced the electron transfer and acted as a mediator in the electrochemical measurements using the redox probe. Figure 5 inset I shows the standard calibration curve obtained from plotting the peak current vs. different concentrations of *O. tsutsugamushi* ssGDNA. A linear dynamic range of 0.05 × 10^2^ pg/µL to 6.3 × 10^2^ pg/µL ssGDNA with a correlation coefficient of R^2^ = 0.92 was recorded. The standard calibration curve in Figure 5 inset II was used to calculate the sensitivity and LOD of the DNA sensor. The sensitivity of the developed sensor was 1228.43 µAcm^−2^ng^−1^ with the detection limit (LOD) of 20 pg/μL ssGDNA of *O. tsutsugamushi*.

### 3.5. Selectivity, Specificity, and Stability of the Fabricated DNA Sensor

**Figure 6:**
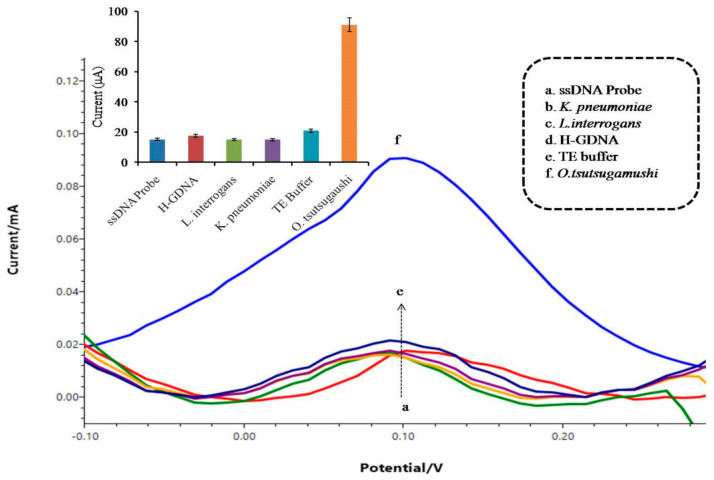
Selectivity of SPPE/GO/ssDNA_probe_/BSA for the detection of *O. tsutsugamushi*. The curve a shows the DPV of SPPE/GO/ssDNA_probe_/BSA, and curves b to f show DPVs of *K. pneumoniae*, *L. interrogans,* Human GDNA (H-GDNA), TE buffer without GDNA, and *O. tsutsugamushi,* respectively.

The selectivity of the DNA sensor was evaluated by comparing the sensor response in terms of peak current with respect to the Probe with the ssGDNA of *O. tsutsugamushi* and other bacterial GDNA (*L. interrogans* and *K. pneumonia)*. As shown in the Figure 6 curve a to g, a negligible difference in peak current was observed with respect to the probe after hybridization with the non-targeted DNA samples. A considerable hike in peak current was observed only when the targeted sample (ssGDNA of *O. tsutsugamushi)* was hybridized with the probe, confirming the selectivity of the sensor. The TE buffer was taken as a blank in the study, and H-GDNA was taken as a negative control.

The specificity of SPPE/GO/BSA/ssDNA_probe_ was evaluated in the presence of a complementary DNA sequence (cDNA) and different numbers of mismatched bases. The specificity was calculated by comparing the peak current values with respect to the probe after hybridization with cDNA and varied mismatched bases, i.e., 1 base, 2 bases, 3 bases, 4 bases, and multiple base mismatched DNA sequences (Table 1).

**Figure 7:**
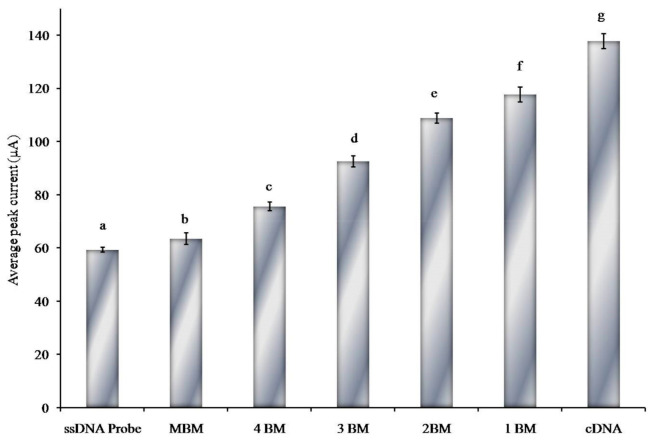
Specificity of SPPE/GO/ssDNA_probe_/BSA for detection of *O. tsutsugamushi*.

As shown in Figure 7, the average peak current value was highest in the case of cDNA (Figure 7g), and a decline in average peak current was observed (Figure 7b–f) with an increase in the number of mismatched bases. Equivalence with respect to the probe was recorded in the case of the multiple base mismatched DNA sequences (Figure 7b), confirming that the sensor is specific only to the complementary sequence.

**Figure 8:**
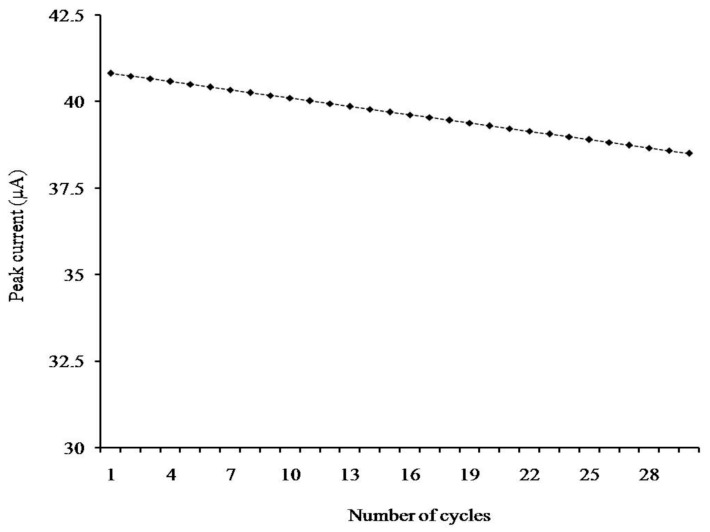
Stability of SPPE/GO/ssDNA_probe_/BSA response for detection of *O. tsutsugamushi*.

The stability of SPPE/GO/BSA/ssDNA_probe_ was evaluated using CV after hybridization with ssGDNA of *O. tsutsugamushi* (Figure 8). The sensor response in terms of peak separation remained unchanged until 30 repetitive cycles with only a 5.7% overall decrease (0.2% decrease per cycle) in peak current with respect to the initial response.

The results show higher stability of the modified paper electrode (SPPE/GO/BSA/ssDNA_probe_).

## 4. Conclusions

In this research work, an electrochemical paper-based DNA sensor (SPPE/GO/BSA/ssDNA_probe_) was designed for selective detection of *O. tsutsugamushi* GDNA. The sensor showed higher sensitivity of 1228.43 µA cm^−2^ ng^−1^ and lower detection limit (LOD) of 20 pg/μL for detection of *O. tsutsugamushi* GDNA. The advantages of the developed method over existing diagnosis methods include higher sensitivity, selectivity, lower detection limit, higher stability, and low cost. The developed assay can replace the existing methods of diagnosis and can become an ideal tool for the detection of the disease at early stages. The simplicity, portability, and quick response (15–20 min) of the DNA sensor enable onsite detection of the disease even outside the laboratory settings. The features of the developed sensor make it an ideal method for the diagnosis of scrub typhus.

## Figures and Tables

**Table 1:** Nucleotide sequences (cDNA and mismatch bases) used in selectivity.

DNA Sample	Mismatch Base Sequences
cDNA	5′ACCCCAGAACCTAAGAACACT 3′
1 base mismatch	5′AGCCCAGAACCTAAGAACACT 3′
2 base mismatch	5′ AGGCCAGAACCTAAGAACACT 3′
3 base mismatch	5′ AGGGCAGAACCTAAGAACACT 3′
4 base mismatch	5′ TGGGCAGAACCTAAGAACACT 3′
Multiple base mismatch	5′ TGGGCACAAGGTAAGTTCAGT 3′
